# Anterior segment inflammation and its association with dry eye parameters following myopic SMILE and FS-LASIK

**DOI:** 10.1080/07853890.2023.2181388

**Published:** 2023-02-23

**Authors:** Jian Zhao, Yuan Li, Tianyun Yu, Wenhao Wang, Mutsvene Tinashe Emmanuel, Qianwen Gong, Liang Hu

**Affiliations:** a National Clinical Research Center for Ocular Diseases, Eye Hospital, Wenzhou Medical University, Wenzhou, P.R. China; b National Engineering Research Center of Ophthalmology and Optometry, Eye Hospital, Wenzhou Medical University, Wenzhou, P.R. China; c New England College of Optometry, Boston, MA, USA; d School of Optometry and Vision Science, University of New South Wales, Sydney, Australia; e Department of Ophthalmology, Affiliated People’s Hospital of Ningbo University, Ningbo, P.R.China

**Keywords:** Dry eye disease, anterior chamber flare, ocular redness, SMILE, FS-LASIK

## Abstract

**Purpose:**

To evaluate dry eye and anterior segment inflammation after small incision lenticule extraction (SMILE) and femtosecond laser-assisted *in situ* keratomileusis (FS-LASIK), and investigate their association.

**Methods:**

This prospective and observational study included 96 eyes from 48 myopic patients. The evaluation was performed at baseline, postoperative day 1, week 1, month 1 and month 3. Outcome measures included anterior chamber flare, bulbar redness (BR), limbal redness (LR), ocular surface disease index (OSDI), tear meniscus height (TMH), the first and average noninvasive breakup time (NIBUT-1, NIBUT-a), fluorescein breakup time (FBUT), corneal fluorescein staining (CFS), and Schirmer I. Generalized estimating equations (GEEs) were applied to explore the correlation between flare and ocular surface parameters.

**Results:**

Flare increased significantly in both groups at day 1 and week 1 and then returned to baseline at month 1. In both groups, BR decreased on day 1 and then gradually increased towards the baseline. In FS-LASIK, LR was lower than baseline at day 1 and month 3. An increase in OSDI was found in the SMILE group on day 1, and in the FS-LASIK group at day 1 to month 1. NIBUT-1 and NIBUT-a decreased significantly on day 1 in both groups. At month 3, NIBUT-a did not return to baseline in FS-LASIK. CFS increased significantly at week 1 in both groups. All parameters were comparable between SMILE and FS-LASIK except for OSDI and NIBUT-a. Time and spherical equivalent showed a correlation with flare.

**Conclusions:**

Both SMILE and FS-LASIK induced elevated anterior chamber flare and dry eye. However, flare might not be considered a factor determining perioperative dry eye.

**Key Messages**
- Dry eye disease is common after corneal refractive surgery. Signs and symptoms of dry eye disease persist longer after FS-LASIK compared with SMILE.
- Both FS-LASIK and SMILE transiently disrupted blood-aqueous barrier integrity, leading to anterior segment inflammation.
- Anterior chamber flare might not be considered a factor explaining perioperative dry eye, other biomarkers remain for future exploration.

## Background

Myopia is one of the leading causes of reversible visual impairment worldwide [1], with a prevalence approximating 80-90% among young adults in East Asia [2]. Myopic corneal laser vision correction (LVC) is widely accepted, and currently, the mainstream of LVC in China includes small incision lenticule extraction (SMILE) and femtosecond laser-assisted *in situ* keratomileusis (FS-LASIK). Dry eye disease (DED) is one of the most frequent complications after LVC [1,3]. Previous studies have shown that DED did not merely cause visual quality impairment post corneal refractive surgery [4], but played a cardinal role in regression [5], thus impairing patient quality of life.

Inflammation is one of the pathogenic factors involved in the vicious cycle of DED [6,7], therefore anterior segment inflammation caused by LVC might be a critical participant in post-LVC DED. Proper evaluation of inflammation may shed some light on the assessment of DED severity, application of anti-inflammatory medication, and improvement in patient satisfaction following LVC. The anterior chamber flare reflects the blood-aqueous barrier integrity during inflammation [8]. Previous studies revealed that flare was sensitive to detect inflammation after LVC and corneal cross-linking [9]. The ocular redness, meanwhile, represents the vasodilatory intensity of the microvasculature in response to inflammation [10]. Limbal hyperemia is more closely related to inflammatory changes in the cornea [11]. However, traditional methods for assessing ocular redness are primarily subjective [12], and it is difficult to grade the limbal and bulbar areas separately. These disadvantages have been overcome by the most recent Keratograph 5 M (Oculus GmbH, Wetzlar, Germany), hence the present study used it for a quantitative assessment of conjunctival hyperemia.

This study set out to prospectively measure anterior segment inflammation (flare and ocular redness) and dry eye metrics before and after SMILE and FS-LASIK, in order to investigate the potential relationship between LVC-related inflammation and dry eye.

## Materials and methods

### Subjects

The study was approved by the Ethics Committee of the Eye Hospital, Wenzhou Medical University (2021-024-K-21-01). Each patient provided informed consent after receiving a detailed explanation of the research. This trial was conducted in compliance with the principles of Declaration of the Helsinki.

Inclusion criteria were preoperative age between 18-40 years; manifest refractive spherical equivalent (MRSE) less than 10.00 diopters (D), astigmatism less than 3.50 D; and refractive status stable for 2 years. All patients stopped wearing rigid contact lenses at least one month prior, and soft contact lens for at least 2 weeks. Patients were excluded if they had any medical condition; recent history of topical or systemic medication use; an active illness of the eye; history of trauma or surgery to the eye; evidence of keratoconus, glaucoma, or vitreoretinal disorders in either eye; and intolerable side effects of postoperative medication.

### Surgical technique

LVC was performed by the same experienced operator (LH). The VisuMax femtosecond laser (Carl Zeiss Meditec AG, Jena, Germany) was utilized during the SMILE procedure. It involved removing a stromal lenticule, with a 110 µm-thick cap. For FS-LASIK, the lamellar flap with a superior hinge was created by a femtosecond laser (Ziemer Ophthalmic Systems AG, Port, Switzerland). The maximum thickness ranged from 90 to 110 µm. Tissue ablation was then performed using an Amaris 750 Hz excimer laser (Schwind eye-tech-solutions, Kleinostheim, Germany). Both groups received one drop of tobramycin/dexamethasone (Tobradex) shortly after surgery. A bandage contact lens (Oasys; Johnson & Johnson Vision, Santa Ana, USA) was placed on the cornea after FS-LASIK surgery. Sodium hyaluronate 0.1% (Hylo-comod) and topical fluorometholone 0.1% were then used 4 times a day for one week. The fluorometholone dosage was tapered each subsequent week until termination one month, while the Hylo-comod dosage remained unaltered.

### Evaluation of inflammatory response

Measurements were performed without any mydriatic agent at baseline and repeated after 1 day, 1 week, 1 month, and 3 months. On day 1, tests were performed after the removal of contact lenses. The laser flare photometer FM-600 (Kowa, Nagoya, Japan) was used to assess anterior chamber inflammation. Five consecutive readings from the lower third of the anterior chamber were recorded under dim ambient illumination. The background scatter should be less than 10%. After excluding the highest and lowest values, the average of the remaining readings was obtained. Keratograph 5 M (Oculus GmbH, Wetzlar, Germany) was used to assess bulbar redness (BR) and limbal redness (LR). Patients were instructed to keep their eyes wide open. Redness was graded from avascular (0) to severely hyperemic (4) based on the area percentage ratio between the vessels and the rest of the analyzed area [13].

### Tests for dry eye

To prevent any effects from eyedrops, the subjects were instructed to discontinue use at least 1 h prior to examination. At each follow-up, the Ocular Surface Disease Index (OSDI) questionnaires [14] were filled out to evaluate dry eye symptoms. Tear meniscus height (TMH), first and average noninvasive tear film break-up time (NIBUT-1, NIBUT-a) were recorded by the Keratograph 5 M. The average of three consecutive measurements was recorded. The fluorescein tear film break-up time (FBUT), corneal fluorescein staining (CFS) score and Schirmer I value were evaluated according to the previously established methods [15]. Given their invasive nature, FBUT, CFS and Schirmer tests were not evaluated on day 1.

### Statistical analysis

Statistical analysis was performed using SPSS, version 26.0 (IBM Corp, NY, USA), and based on data from both of the participant's eyes. Data were evaluated for normality using the Shapiro-Wilk test, and were expressed as mean ± standard deviation. Using the Mann–Whitney U test or the independent-sample *t* test, comparisons of the demographic data were made (gender differences were analyzed using the chi-square test). To compare variables between time and group, two-way repeated measures ANOVA or generalized estimating equations (GEEs) were used, and multiple comparisons were handled using the Bonferroni correction. To investigate the factors correlated with flare, significant variables were included in the GEEs after collinearity diagnostic tests. *p* < 0.05 was considered statistically significant. For repeated measures analysis, the sample size was calculated using PASS 15.0 software (NCSS, Utah, USA). The calculation (desired power: 0.90; *α* = 0.05) using the flare data obtained in our preliminary study indicated that fourteen eyes in each group were required to detect the within-group difference and allow for a 20% dropout rate during follow-up.

## Results

The study was conducted between February 2021 to September 2021, with 48 patients (86%) remaining at month 3. The eyes with subconjunctival hemorrhage after LVC were excluded from the redness analysis until the bleeding was completely absorbed. At baseline, the demographic and clinical parameters were comparable between the two groups (Table 1 and Supplementary Table 1). Refractive outcomes were similar between SMILE and FS-LASIK (Supplementary Figure 1).

**Table 1.** Demographic characteristics between groups.

Variable	SMILE	FS-LASIK	Statistics	*P*
Gender (male/female)	14/10	12/12	0.336	0.562^a^
Age (years)	23.38 ± 6.21	25.33 ± 5.85	–1.345	0.179^c^
MRSE (D)	–5.16 ± 1.45	–5.38 ± 1.69	–0.290	0.772^c^
Cylinder (D)	–0.90 ± 0.53	–0.96 ± 0.90	–0.917	0.359^c^
HADS	5.13 ± 3.42	6.42 ± 4.48	–1.123	0.267^b^

MRSE: manifest refraction spherical equivalent; HADS: hospital anxiety and depression scale.

^a^Represents chi-square test.

^b^Represents independent-samples *t* test.

^c^Represents Mann–Whitney U test.

### Flare and ocular redness

**Figure 1.**
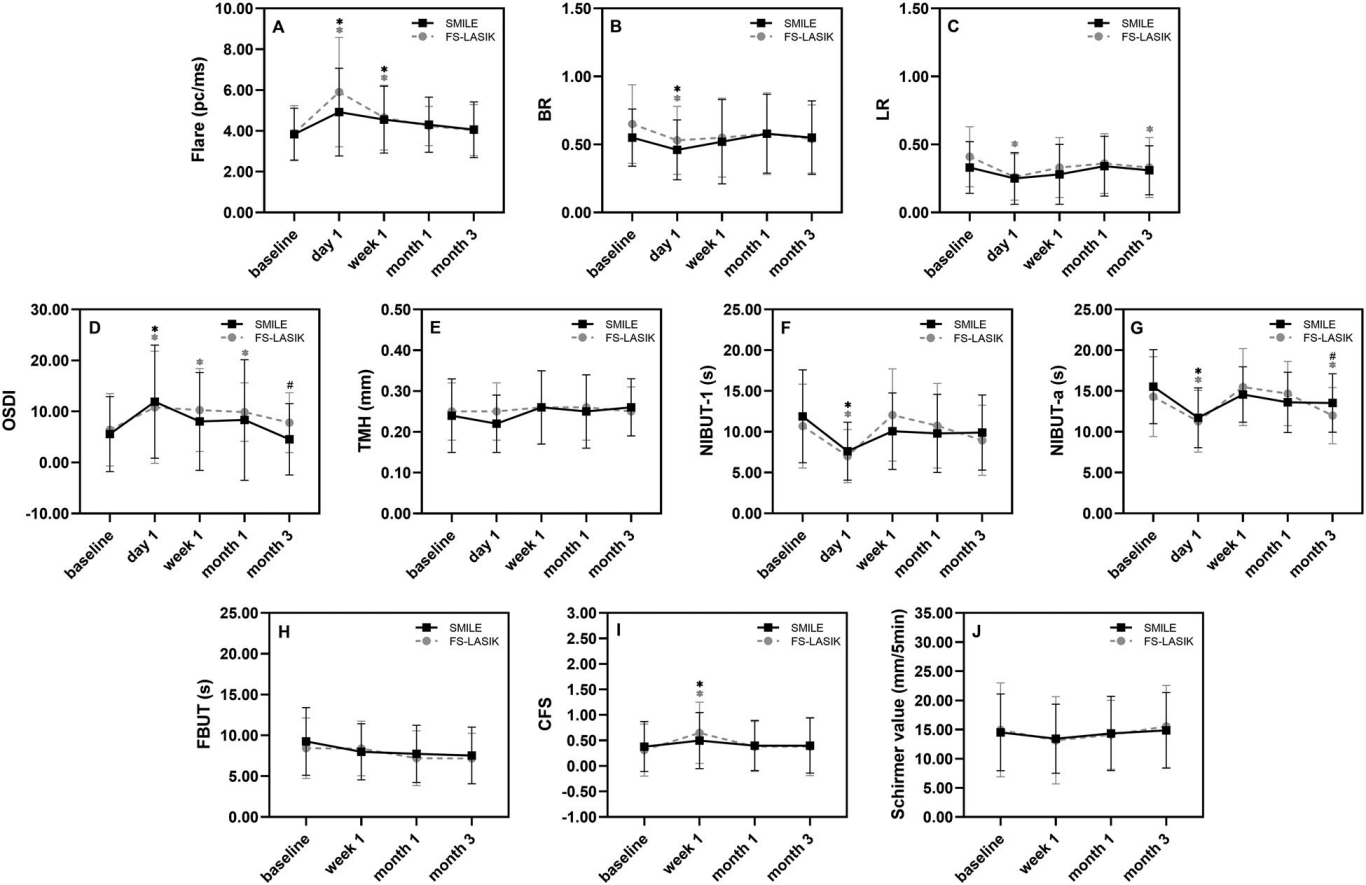
Time-dependent change of inflammatory markers and dry eye metrics. BR: bulbar redness, LR: limbal redness, OSDI: ocular surface disease index, TMH: tear meniscus height, NIBUT-1: noninvasive tear film break-up time first, NIBUT-a: noninvasive tear film break-up time average, FBUT: fluorescein tear film break-up time, CFS: corneal fluorescein staining score using the Oxford scheme; *statistically significant difference from preoperative value, ^#^statistically significant difference between groups.

As shown in Figure 1, flare significantly increased following SMILE and FS-LASIK, at day 1 (*p* < 0.001) and week 1 (*p* = 0.017), then returned to normal at month 1 and month 3. There was no significant difference between the groups from baseline to month 3. BR decreased significantly after either SMILE or FS-LASIK (*p* < 0.001) on day 1, then showed a steady increase towards the baseline level. In the SMILE group, LR had no significant change at any timepoint compared to baseline, whereas it decreased significantly in the FS-LASIK group at day 1 and month 3 compared to baseline. Inter-group comparisons indicated that ocular redness was comparable between the groups.

### Dry eye parameters

OSDI increased significantly on day 1 after SMILE, compared with the baseline (*p* < 0.001) (Figure 1). For FS-LASIK, OSDI increased significantly at day 1 (*p* = 0.002), at week 1 (*p* < 0.001), and at month 1 (*p* = 0.007). OSDI was higher in the FS-LASIK group at month 3 compared with SMILE (*p* = 0.012). NIBUT-1 and NIBUT-a decreased significantly at day 1 in both groups (*p* < 0.001) and returned to preoperative levels at week 1. However, NIBUT-a decreased at month 3 in the FS-LASIK group (*p* = 0.031) and was higher in the SMILE group (*p* = 0.032). CFS increased significantly (*p* = 0.029) at week 1 in the two groups, then decreased at month 1. TMH, FBUT and Schirmer scores were not statistically different at all follow-up points. Of note, no inter-group difference was observed in terms of these parameters.

### Correlation analysis

Table 2 shows the factors that might correlate with flare. BR (*β* = −0.416, *p* = 0.243), LR (*β* = −0.352, *p* = 0.390), and other parameters were excluded after univariate analysis. When the SMILE group was selected as reference, the GEE model revealed no significant difference between SMILE and FS-LASIK (*β* = 0.202, *p* = 0.245). NIBUT-1 and NIBUT-a showed collinearity, therefore we used GEE1 and GEE2, displaying consistent outcomes. When compared to the baseline, the flare showed a significant increase on day 1, week 1 and month 1. The GEE test results indicated that MRSE correlated negatively with flare (*p* < 0.001), whereas neither NIBUT-1 nor NIBUT-a correlated with flare (*p >* 0.05).

**Table 2.** Factors correlated with flare during all follow-up time points.

GEE1	β	*P*	GEE2	*β*	*P*
Intercept	2.423	<0.001	Intercept	2.437	<0.001
Time 0	Reference	–	Time 0	Reference	–
Time 1	1.493	<0.001	Time 1	1.512	<0.001
Time 2	0.731	<0.001	Time 2	0.735	<0.001
Time 3	0.396	0.028	Time 3	0.402	0.026
Time 4	0.161	0.353	Time 4	0.165	0.349
MRSE (D)	−0.302	<0.001	MRSE (D)	−0.299	<0.001
NIBUT-1 (s)	–0.013	0.357	NIBUT-a (s)	–0.010	0.558

NIBUT-1 and NIBUT-a showed collinearity, thus we took GEE respectively in Table 2: 2-1 (Dependent Variable: Flare. Model: (Intercept), Time, MRSE, NIBUT-1) and 2-2 (Dependent Variable: Flare. Model: (Intercept), Time, MRSE, NIBUT-a). Time 0: baseline; Time 1: day 1; Time 2: week 1; Time 3: month 1; Time 4: month 3; MRSE: Manifest refraction spherical equivalent; NIBUT-1: noninvasive tear film break-up time first; NIBUT-a: noninvasive tear film break-up time average.

## Discussion

Dry eye is one of the most common adverse effects of LVC, and it is mostly induced by decreased tear production due to corneal nerve damage and inflammation [1]. Detection and quantification of inflammatory DED biomarkers can help customize DED management post-LVC, since the associated inflammation can have ramifications for corneal wound healing and refractive outcomes [16,17]. Current clinical methods of assessing inflammation in DED include observing conjunctival hyperemia, mucus alterations, and epithelial vital staining, even though these assessments are subjective and fall short of precision [12]. The anterior chamber flare, a marker of inflammation activity in the anterior chamber, was found to increase significantly in the aqueous humor of patients with DED [8], or after various types of corneal refractive surgeries [18–20]. We hypothesized that there might be a potential association between inflammatory biomarkers (anterior chamber flare or ocular redness) and dry eye severity in patients who have undergone LVC. Hence, the current study was designed to investigate the role of flare and ocular redness in evaluating perioperative dry eye. This is the first study to quantify changes of anterior chamber flare and ocular redness following SMILE. The primary findings were an increase in flare and a worsening of dry eye parameters after surgery, with the majority of patients returning to baseline by month 3.

Traditionally, LVC procedures were aimed at compensating for corneal refractive power, therefore visual function and cornea were assessed extensively to confirm the safety and efficacy. It should be noted, however, that little is known regarding the impact of LVC on the aqueous humour, vitreous, and retina. Early studies [18–26] observed anterior chamber flare in patients who underwent photorefractive keratectomy (PRK) and LASIK, with contradictory findings (Table 3). In most investigations that revealed an increase in anterior chamber flare, the blood-aqueous barrier breach was transitory and reversible. Thus, PRK or LASIK could be considered safe refractive procedures in this regard, which explains why few studies were conducted later to investigate flare after LVC. However, two new forms of LVC have been introduced: FS-LASIK and SMILE. To obtain a better understanding of anterior chamber changes after these procedures, it is necessary to investigate aqueous flare following these two procedures. In this study, both groups showed an increase in a flare on day 1 to week 1 after surgery before recovering to baseline status at month 1, with no significant differences between the groups. El-Harazi et al. [18] found that LASIK induced a significant increase in the flare on day 1, and then returned to baseline levels by day 7. Tomas-Barberan et al. [19] reported a prominent flare elevation at 5 days and 12 days post PRK in the 1-month group. Vita et al. [20] found no statistically significant flare increase on the first day after PRK or LASIK. Comparatively, this study obtained a lower flare value. The application of femtosecond laser in LVC may cause less damage to the cornea during flap or lenticular creation. Interestingly, SMILE also caused anterior chamber inflammation, and demonstrated a similar changing trend in the flare with FS-LASIK.

**Table 3.** Brief summary of flare after laser vision correction.

Author	Laser flare meter	Group	Number of eyes	Follow-up time	Main findings
Tomas-Barberan et al. [19]	Kowa FM 500	PRK (1-week)	10	1 week	Aqueous flare increased significantly in both groups. Topical corticosteroids did not prevent breakdown of the blood-aqueous barrier after operation.
		PRK (1-month)	15	1 month	
Vita et al. [20]	Kowa FM 500	PRK	15	15 days	Neither PRK or LASIK induced statistically significant increased flare. PTK resulted in significant elevations in flare.
		LASIK	11	15 days	
		PTK	10	15 days	
Pérez-Santonja et al. [21]	Kowa FC 1000	LASIK	20	3 months	LASIK did not induce inflammation in the anterior chamber.
Tomas-Barberan et al. [22]	Kowa FM 500	PRK (diclofenac)	20	3 days	Although not statistically significant, there is a tendency toward less flare elevation in the anterior chamber in eyes treated with topical diclofenac and dexamethasone compared to placebo.
		PRK (dexamethasone)	20	3 days	
		PRK (placebo)	20	3 days	
Nguyen et al. [23]	Kowa FC 1000	PRK	31	3 months	No significant increase of aqueous flare was found.
Pisella et al. [24]	Kowa FM 500	PRK	N/A	21 days	Aqueous flare increased significantly in both groups. The increase probably correlated with the depth of photoablation.
		LASIK	N/A	21 days	
El-Harazi et al. [18]	Kowa FM 500	LASIK	43	7 days	Despite the topical corticosteroids used, a significant increase in anterior chamber flare occurred.
Sen et al. [25]	Kowa FM 500	LASIK	10	2 weeks	LASIK induced a short-term postoperative increase in aqueous flare.
Özbilen et al. [26]	Kowa FM 700	FS-LASIK	30	3 months	FS-LASIK caused minimal inflammation in the anterior chamber.

PRK: photorefractive keratectomy; LASIK: laser *in situ* keratomileusis; PTK: phototherapeutic keratectomy.

The conjunctival microvasculature is sensitive to a wide range of non-infectious etiologies, resulting in a localized inflammatory response and a reddish appearance of the conjunctival tissue [10]. Following LVC, it was found that ocular redness decreased in post-FS-LASIK or SMILE eyes. Studies has proven the efficacy of corticosteroids in alleviating conjunctival hyperemia [27]. In this study, the topical use of corticosteroids contributed to the management of conjunctival hyperemia. Furthermore, patients were advised to take adequate rest within 24 h after LVC. With less oxygen consumption, conjunctival vasoconstriction led to decreased BR and LR on day 1. There was no difference in BR or LR between SMILE and FS-LASIK. Results from Luft et al. [28] also showed mild and comparable keratocyte proliferation, keratocyte apoptosis, and infiltration of inflammatory cells between FS-LASIK and SMILE in *ex vivo* human eyes. In this study, the ocular redness did not achieve clinical relevance and showed no correlation with flare, which could be attributed to the anti-inflammatory medication on the ocular surface.

Our results showed no significant difference between groups in terms of TMH, NIBUT-1, FBUT, CFS, and Schirmer scores at each follow-up point. However, SMILE had less impact on tear film stability. During the 3-month period, patients experienced dry eye symptoms, which lasted shorter than the FS-LASIK group. According to the NIBUT and CFS findings, both SMILE and FS-LASIK caused tear film instability and corneal fluorescein staining, but NIBUT-a did not return to baseline in the FS-LASIK group at month 3. These findings support prior studies that proved the superiority of SMILE over FS-LASIK in terms of dry eye [29–32].

This study found no link between anterior chamber flare and OSDI, BUTs and other dry eye parameters, similar to the results of Aghaei et al. [8] who indicated no correlation between flare and OSDI or FBUT in dry eye patients. A few studies have analyzed tear inflammatory mediators or neurotrophins in post-LVC eyes and compared them with dry eye metrics. González-García et al. [33] comprehensively evaluated the alteration in tear molecules after advanced surface ablation and found that the OSDI had no significant correlation with cytokines. The small sample size could affect their experimental results. Gao et al. [34] characterized the early change in tear inflammatory mediators following LVC, interleukin-6 levels were found to correlate positively with OSDI in both the SMILE and FS-LASIK group, and with CFS in the FS-LASIK group. The increase in interleukin-6 could be explained by the corneal inflammation resulting from LVC. The nerve growth factor levels correlated positively with OSDI and CFS in the FS-LASIK group, but not in SMILE. This might be because the minor incision of SMILE failed to stimulate the nerve growth factor expression. Although the administration of eye drops may have obscured the potential link between inflammation and dry eye in our study, it was unrealistic to avoid the use of topical medication due to ethical concerns. Based on the GEE models, flare showed a negative correlation with MRSE, suggesting that eyes with a higher degree of myopia preoperatively tend to experience more inflammation in the anterior chamber after LVC [19]. The reason may be that more stromal tissue was ablated in high myopic eyes. These eyes received a greater number of laser pulses and required longer surgical time, resulting in a more severe inflammatory response.

The present study had certain limitations. Firstly, the follow-up time was short. However, the flare returned to normal at month 1, and ocular redness did not increase significantly in both groups; 3 months seemed enough to observe anterior segment inflammation. Secondly, despite ocular redness and flare being convenient and clinically feasible indices, the present study did not test tear inflammatory factors, ongoing studies by our team are currently exploring alteration in tear cytokines and neuropeptides following LVC.

In conclusion, SMILE and FS-LASIK were generally comparable with regard to postoperative inflammation and dry eye. However, dry eye symptoms and signs persisted for a longer time following FS-LASIK. Although higher anterior chamber flare may not be related to post-LVC dry eye, discovering acceptable inflammatory biomarkers for post-LVC dry eye, whether fundamentally or clinically, remains a relatively undiscovered field deserving of future investigation.

## Commercial relationships

All of the authors have no commercial relationships.

## Supplementary Material

Supplemental Material

Supplemental Material

## Data Availability

The data that support the findings of this study are available from the corresponding authors (LH & QWG) upon reasonable request.

## References

[1] Kim T, Alió Del Barrio J, Wilkins M, et al. Refractive surgery. Lancet. 2019;393(10185):2085–2098. 31106754 10.1016/S0140-6736(18)33209-4

[2] Wu PC, Huang HM, Yu HJ, et al. Epidemiology of myopia. Asia Pac J Ophthalmol 2016;5(6):386–393. 10.1097/APO.0000000000000236 27898441

[3] Sharma B, Soni D, Saxena H, et al. Impact of corneal refractive surgery on the precorneal tear film. Indian J Ophthalmol. 2020;68(12):2804–2812. 33229655 10.4103/ijo.IJO_2296_19 PMC7856956

[4] Murakami Y, Manche E. Prospective, randomized comparison of self-reported postoperative dry eye and visual fluctuation in LASIK and photorefractive keratectomy. Ophthalmology. 2012;119(11):2220–2224. 22892151 10.1016/j.ophtha.2012.06.013

[5] Albietz J, Lenton L, McLennan S. Chronic dry eye and regression after laser in situ keratomileusis for myopia. J Cataract Refract Surg. 2004;30(3):675–684. 15050267 10.1016/j.jcrs.2003.07.003

[6] Baudouin C, Irkeç M, Messmer EM, et al. Clinical impact of inflammation in dry eye disease: proceedings of the ODISSEY group meeting. Acta Ophthalmol. 2018;96(2):111–119. 28390092 10.1111/aos.13436 PMC5836968

[7] Bron A, de Paiva C, Chauhan S, et al. TFOS DEWS II pathophysiology report. Ocul Surf. 2017;15(3):438–510. 28736340 10.1016/j.jtos.2017.05.011

[8] Aghaei H, Kheirkhah A, Es’ Haghi A, et al. Disruption of blood-aqueous barrier in dry eye disease. Ocul Surf. 2021;19:266–269. 33065256 10.1016/j.jtos.2020.10.002

[9] Hedayatfar A, Hashemi H, Aghaei H, et al. Subclinical inflammatory response: accelerated versus standard corneal cross-linking. Ocul Immunol Inflamm. 2019;27(3):513–516. 29333909 10.1080/09273948.2017.1420201

[10] Singh R, Liu L, Anchouche S, et al. Ocular redness – I: etiology, pathogenesis, and assessment of conjunctival hyperemia. Ocul Surf. 2021;21:134–144. 34010701 10.1016/j.jtos.2021.05.003 PMC8328962

[11] Ohta K, Wiggert B, Taylor A, et al. Effects of experimental ocular inflammation on ocular immune privilege. Invest Ophthalmol Vis Sci. 1999;40(9):2010–2018. 10440255

[12] Rolando M, Barabino S. Are there clinical ways to assess inflammation in dry eye disease? Ocul Immunol Inflamm. 2021;29(6):1183–1189. 34227903 10.1080/09273948.2021.1916540

[13] Wu S, Hong J, Tian L, et al. Assessment of bulbar redness with a newly developed keratograph. Optom Vis Sci. 2015;92(8):892–899. 26099055 10.1097/OPX.0000000000000643

[14] Schiffman R, Christianson M, Jacobsen G, et al. Reliability and validity of the ocular surface disease index. Arch Ophthalmol. 2000;118(5):615–621. 10815152 10.1001/archopht.118.5.615

[15] Li Y, Li S, Zhou J, et al. Relationship between lipid layer thickness, incomplete blinking rate and tear film instability in patients with different myopia degrees after small-incision lenticule extraction. PLOS One. 2020;15(3):e0230119. 32163466 10.1371/journal.pone.0230119 PMC7067460

[16] Kumar NR, Khamar P, Shetty R, et al. Identification of novel predictive factors for post surgical corneal haze. Sci Rep. 2019;9(1):16980. 31740714 10.1038/s41598-019-53123-3 PMC6861263

[17] Shetty R, Sethu S, Chevour P, et al. Lower vitamin D level and distinct tear cytokine profile were observed in patients with mild dry eye signs but exaggerated symptoms. Transl Vis Sci Technol. 2016;5(6):16. 10.1167/tvst.5.6.16 PMC5156440 27980879

[18] El-Harazi S, Chuang A, Yee R. Assessment of anterior chamber flare and cells after laser in situ keratomileusis. J Cataract Refract Surg. 2001;27(5):693–696. 11377897 10.1016/s0886-3350(01)00798-2

[19] Tomas-Barberan S, Fagerholm P. Anterior chamber flare after photorefractive keratectomy. J Refract Surg. 1996;12(1):103–107. 8963797 10.3928/1081-597X-19960101-19

[20] Vita R, Campos M, Belfort R, et al. Alterations in blood-aqueous barrier after corneal refractive surgery. Cornea. 1998;17(2):158–162. 9520191 10.1097/00003226-199803000-00007

[21] Pérez-Santonja JJ, Sakla HF, Cardona C, et al. Subclinical inflammation after laser in situ keratomileusis. J Cataract Refract Surg. 1998;24(8):1059–1063. 9719964 10.1016/s0886-3350(98)80098-9

[22] Tomas-Barberan S, Fagerholm P. Influence of topical treatment on epithelial wound healing and pain in the early postoperative period following photorefractive keratectomy. Acta Ophthalmol Scand. 1999;77(2):135–138. 10321525 10.1034/j.1600-0420.1999.770203.x

[23] Nguyen NX, Seitz B, Langenbucher A, et al. Quantification of blood-aqueous barrier breakdown after photorefractive keratectomy for myopia. Graefes Arch Clin Exp Ophthalmol. 1999;237(2):113–116. 9987627 10.1007/s004170050205

[24] Pisella PJ, Albou-Ganem C, Bourges JL, et al. Evaluation of anterior chamber inflammation after corneal refractive surgery. Cornea. 1999;18(3):302–305. 10336033 10.1097/00003226-199905000-00011

[25] Sen HN, Uusitalo R, Laatikainen L. Subclinical inflammation after laser in situ keratomileusis in corneal grafts. J Cataract Refract Surg. 2002;28(5):782–787. 11978455 10.1016/s0886-3350(02)01239-7

[26] Özbilen KT, Altinkurt E, Ceylan NA, et al. Effect of myopic femtosecond laser-assisted LASIK on anterior chamber inflammation (flare values) and corneal endothelium: a prospective before and after study. J Ophthalmol. 2021;2021:2395028. 34868671 10.1155/2021/2395028 PMC8642012

[27] Singh R, Liu L, Yung A, et al. Ocular redness – II: progress in development of therapeutics for the management of conjunctival hyperemia. Ocul Surf. 2021;21:66–77. 34000363 10.1016/j.jtos.2021.05.004 PMC8328932

[28] Luft N, Schumann R, Dirisamer M, et al. Wound healing, inflammation, and corneal ultrastructure after SMILE and femtosecond laser-assisted LASIK: a human *ex vivo* study. J Refract Surg. 2018;34(6):393–399. 29889292 10.3928/1081597X-20180425-02

[29] Denoyer A, Landman E, Trinh L, et al. Dry eye disease after refractive surgery: comparative outcomes of small incision lenticule extraction versus LASIK. Ophthalmology. 2015;122(4):669–676. 25458707 10.1016/j.ophtha.2014.10.004

[30] Xia L, Zhang J, Wu J, et al. Comparison of corneal biological healing after femtosecond LASIK and small incision lenticule extraction procedure. Curr Eye Res. 2016;41(9):1202–1208. 26833247 10.3109/02713683.2015.1107590

[31] Ganesh S, Gupta R. Comparison of visual and refractive outcomes following femtosecond laser- assisted lasik with smile in patients with myopia or myopic astigmatism. J Refract Surg. 2014;30(9):590–596. 25250415 10.3928/1081597X-20140814-02

[32] Xu Y, Yang Y. Dry eye after small incision lenticule extraction and LASIK for myopia. J Refract Surg. 2014;30(3):186–190. 24763723 10.3928/1081597X-20140219-02

[33] González-García M, Murillo G, Pinto-Fraga J, et al. Clinical and tear cytokine profiles after advanced surface ablation refractive surgery: a six-month follow-up. Exp Eye Res. 2020;193:107976. 32081669 10.1016/j.exer.2020.107976

[34] Gao S, Li S, Liu L, et al. Early changes in ocular surface and tear inflammatory mediators after small-incision lenticule extraction and femtosecond laser-assisted laser in situ keratomileusis. PLoS One. 2014;9(9):e107370. 25211490 10.1371/journal.pone.0107370 PMC4161422

