# Supplementary material for: Anterior segment inflammation and its association with dry eye parameters following myopic SMILE and FS-LASIK
Source: Ann Med. 2023 Feb 23;55(1):689–95. doi: 10.1080/07853890.2023.2181388 (PMC9970216; doi:10.1080/07853890.2023.2181388)
Supplement: Supplemental Material [file IANN_A_2181388_SM7815.docx]

**Supplementary Table 1.** Changes in flare and ocular surface parameters over time.

|  | SMILE (n=48) | |  | FS-LASIK (n=48) | |  |  |
| --- | --- | --- | --- | --- | --- | --- | --- |
| Variable | Mean | Std. Dev. | P | Mean | Std. Dev. | P | P’ |
| Flare (pc/ms) |  |  |  |  |  |  |  |
| Time 0 | 3.84 | 1.27 | - | 3.89 | 1.35 | - | 1.000 |
| Time 1 | 4.92 | 2.15 | <0.001 | 5.90 | 2.68 | <0.001 | 1.000 |
| Time 2 | 4.55 | 1.64 | 0.017 | 4.65 | 1.59 | 0.017 | 1.000 |
| Time 3 | 4.30 | 1.35 | 1.000 | 4.24 | 0.96 | 1.000 | 1.000 |
| Time 4 | 4.06 | 1.36 | 1.000 | 4.04 | 1.25 | 1.000 | 1.000 |
| BR |  |  |  |  |  |  |  |
| Time 0 | 0.55 | 0.21 | - | 0.65 | 0.29 | - | 1.000 |
| Time 1 | 0.46 | 0.22 | <0.001 | 0.53 | 0.25 | <0.001 | 1.000 |
| Time 2 | 0.52 | 0.31 | 0.631 | 0.55 | 0.29 | 0.631 | 1.000 |
| Time 3 | 0.58 | 0.29 | 1.000 | 0.58 | 0.30 | 1.000 | 1.000 |
| Time 4 | 0.55 | 0.27 | 0.537 | 0.54 | 0.25 | 0.537 | 1.000 |
| LR |  |  |  |  |  |  |  |
| Time 0 | 0.33 | 0.19 | - | 0.41 | 0.22 | - | 0.055 |
| Time 1 | 0.25 | 0.19 | 0.070 | 0.26 | 0.17 | <0.001 | 0.705 |
| Time 2 | 0.28 | 0.22 | 0.567 | 0.33 | 0.22 | 0.486 | 0.105 |
| Time 3 | 0.34 | 0.22 | 1.000 | 0.36 | 0.22 | 0.542 | 0.610 |
| Time 4 | 0.31 | 0.18 | 1.000 | 0.33 | 0.22 | 0.037 | 0.764 |
| OSDI |  |  |  |  |  |  |  |
| Time 0 | 5.57 | 7.35 | - | 6.42 | 7.10 | - | 0.559 |
| Time 1 | 11.93 | 11.07 | <0.001 | 10.81 | 11.01 | 0.002 | 0.614 |
| Time 2 | 8.06 | 9.60 | 0.067 | 10.28 | 8.14 | <0.001 | 0.215 |
| Time 3 | 8.34 | 11.82 | 0.612 | 9.87 | 5.73 | 0.007 | 0.414 |
| Time 4 | 4.54 | 7.00 | 1.000 | 7.81 | 5.86 | 0.722 | 0.012 |
| TMH (mm) |  |  |  |  |  |  |  |
| Time 0 | 0.24 | 0.09 | - | 0.25 | 0.07 | - | 1.000 |
| Time 1 | 0.22 | 0.07 | 0.283 | 0.25 | 0.07 | 0.283 | 1.000 |
| Time 2 | 0.26 | 0.09 | 1.000 | 0.26 | 0.09 | 1.000 | 1.000 |
| Time 3 | 0.25 | 0.09 | 1.000 | 0.26 | 0.08 | 1.000 | 1.000 |
| Time 4 | 0.26 | 0.07 | 1.000 | 0.25 | 0.06 | 1.000 | 1.000 |
| NIBUT-1 (s) |  |  |  |  |  |  |  |
| Time 0 | 11.89 | 5.70 | - | 10.70 | 5.15 | - | 0.278 |
| Time 1 | 7.62 | 3.55 | <0.001 | 7.02 | 3.26 | <0.001 | 0.388 |
| Time 2 | 10.08 | 4.69 | 0.502 | 12.06 | 5.66 | 1.000 | 0.060 |
| Time 3 | 9.80 | 4.79 | 0.216 | 10.74 | 5.18 | 1.000 | 0.351 |
| Time 4 | 9.91 | 4.61 | 0.517 | 8.95 | 4.30 | 0.502 | 0.285 |
| NIBUT-a (s) |  |  |  |  |  |  |  |
| Time 0 | 15.53 | 4.54 | - | 14.30 | 4.89 | - | 0.208 |
| Time 1 | 11.72 | 3.66 | < 0.001 | 11.29 | 3.79 | < 0.001 | 0.574 |
| Time 2 | 14.57 | 3.39 | 1.000 | 15.48 | 4.72 | 1.000 | 0.281 |
| Time 3 | 13.61 | 3.70 | 0.171 | 14.68 | 3.95 | 1.000 | 0.172 |
| Time 4 | 13.53 | 3.58 | 0.108 | 11.98 | 3.45 | 0.031 | 0.032 |
| FBUT (s) |  |  |  |  |  |  |  |
| Time 0 | 9.26 | 4.14 | - | 8.42 | 3.71 | - | 1.000 |
| Time 2 | 7.99 | 3.44 | 1.000 | 8.39 | 3.34 | 1.000 | 1.000 |
| Time 3 | 7.73 | 3.50 | 0.124 | 7.20 | 3.35 | 0.124 | 1.000 |
| Time 4 | 7.54 | 3.47 | 0.051 | 7.17 | 3.09 | 0.051 | 1.000 |
| CFS |  |  |  |  |  |  |  |
| Time 0 | 0.38 | 0.49 | - | 0.31 | 0.51 | - | 1.000 |
| Time 2 | 0.50 | 0.55 | 0.029 | 0.65 | 0.60 | 0.029 | 1.000 |
| Time 3 | 0.40 | 0.49 | 1.000 | 0.38 | 0.49 | 1.000 | 1.000 |
| Time 4 | 0.40 | 0.54 | 1.000 | 0.38 | 0.57 | 1.000 | 1.000 |
| Schirmer (mm/5min) |  |  |  |  |  |  |  |
| Time 0 | 14.52 | 6.58 | - | 14.94 | 8.05 | - | 1.000 |
| Time 2 | 13.44 | 5.92 | 1.000 | 13.15 | 7.48 | 1.000 | 1.000 |
| Time 3 | 14.33 | 6.35 | 1.000 | 14.10 | 5.91 | 1.000 | 1.000 |
| Time 4 | 14.87 | 6.47 | 1.000 | 15.52 | 7.04 | 1.000 | 1.000 |

BR: bulbar redness, LR: limbal redness, OSDI: ocular surface disease index, TMH: tear meniscus height, NIBUT-1: noninvasive tear film break-up time first, NIBUT-a: noninvasive tear film break-up time average, FBUT: fluorescein tear film break-up time, CFS: corneal fluorescein staining; Time 0: baseline, Time 1: day 1, Time 2: week 1, Time 3: month 1, Time 4: month 3; P < 0.05 is considered statistically significant compared with baseline, while P’ < 0.05 is considered statistically significant between groups.
