# Supplementary figures and images for: Anterior segment inflammation and its association with dry eye parameters following myopic SMILE and FS-LASIK
Source: Ann Med. 2023 Feb 23;55(1):689–95. doi: 10.1080/07853890.2023.2181388 (PMC9970216; doi:10.1080/07853890.2023.2181388)

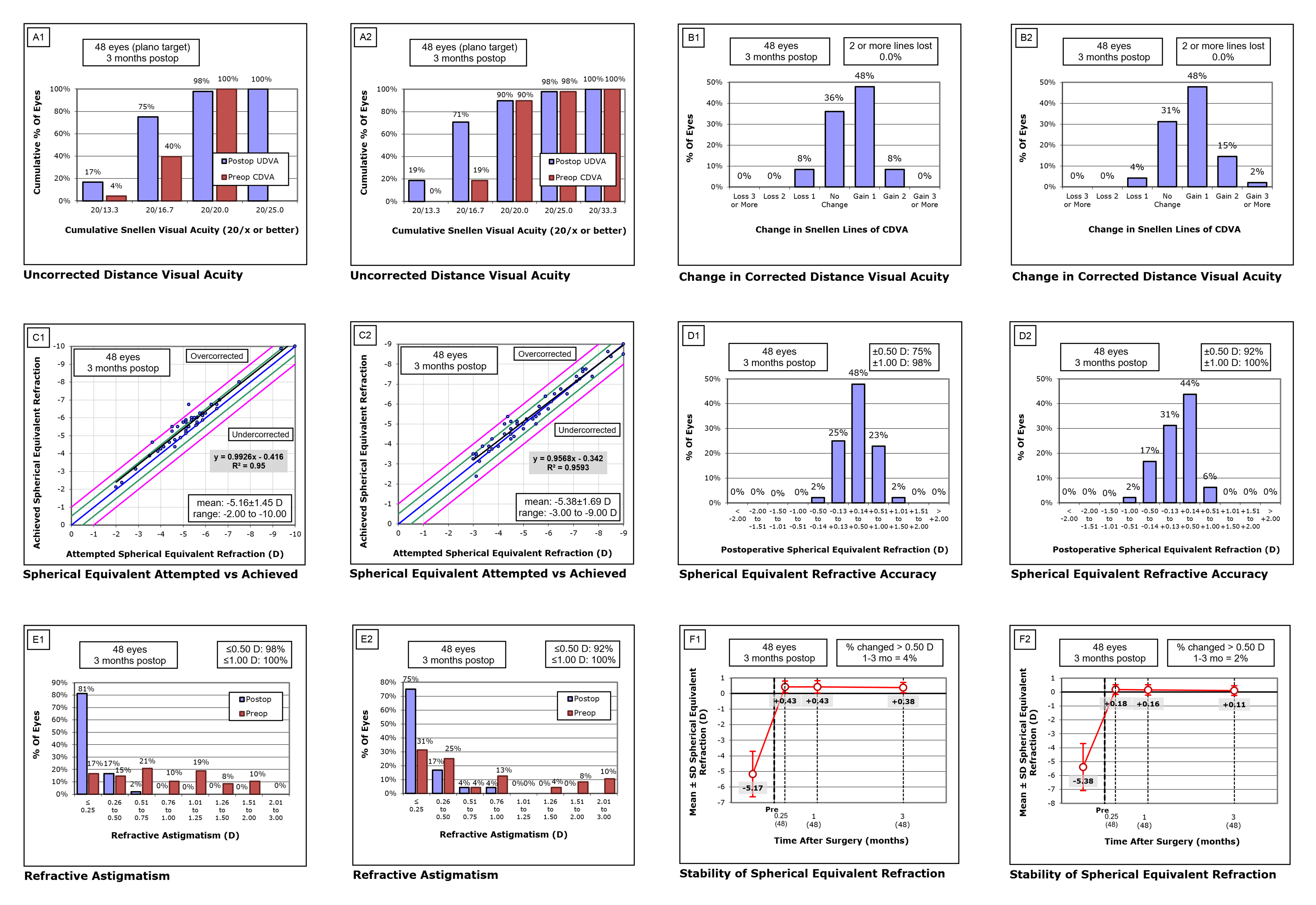

Supplement: Supplemental Material [file IANN_A_2181388_SM7804.tif]
